# 

**Published:** 1902-01-15

**Authors:** 


### CONTENTS.

Some Remarks on Extracting, from an Orthodontist's Point of View, - - *By Milton T. Watson, D.D.S.* 1

Diagnosis in Dental Practice, *By J. Taft, M.D., D.D.S.* 8

Dental Disease an Etiological Factor in Affections of the Eye, - - - - *By Cundell, Juler, M.D.* 23

The President's Address, - *By H. F. Harvey, D.D.S.* 25

Hæmophilia, - - - *By S. D. Ruggles, D.D.S.* 27

Oral Antisepsis in Relation to "Preventive Medicine," *By C. M. Wright, D.D.S.* 39

The Young Dentist Has Come - - - - - - - 42

Editorial, - - - - - - - - - - - 45

Announcements, - - - - - - - - - 47

Bibliography, - - - - - - - - - - 47

Indents, - - - - - - - - - - - 50

